# Systematic literature review and validity evaluation of the Expanded Disability Status Scale (EDSS) and the Multiple Sclerosis Functional Composite (MSFC) in patients with multiple sclerosis

**DOI:** 10.1186/1471-2377-14-58

**Published:** 2014-03-25

**Authors:** Sandra Meyer-Moock, You-Shan Feng, Mathias Maeurer, Franz-Werner Dippel, Thomas Kohlmann

**Affiliations:** 1 Institute for Community Medicine, University Medicine Greifswald, Walther-Rathenau-Strasse 48, 17475 Greifswald, Germany; 2 Department of Neurology, Caritas Krankenhaus, Bad Mergentheim, Germany; 3 Department of Internal Medicine, Neurology and Dermatology, University of Leipzig, Leipzig, Germany

**Keywords:** Multiple sclerosis, Expanded Disability Status Scale (EDSS), Multiple Sclerosis Functional Composite (MSFC), Psychometric properties, Validity, Reliability, Sensitivity of change

## Abstract

**Background:**

There are a number of instruments that describe severity and progression of multiple sclerosis and they are increasingly used as endpoints to assess the effectiveness of therapeutic interventions. We examined to what extent the psychometric properties of two accepted instruments – EDSS and MSFC – meet methodological standards and the value they have in clinical trials.

**Methods:**

We conducted a systematic literature search in relevant databases [MEDLINE (PubMed), ISI Web of Science, EMBASE, PsycINFO & PSYNDEX, CINAHL] yielding 3,860 results. Relevant full-text publications were identified using abstract and then full-text reviews, and the literature was reviewed.

**Results:**

For evaluation of psychometric properties (validity, reliability, sensitivity of change) of EDSS and MSFC, 120 relevant full-text publications were identified, 54 of them assessed the EDSS, 26 the MSFC and 40 included both instruments. The EDSS has some documented weaknesses in reliability and sensitivity to change. The main limitations of the MSFC are learning effects and the z-scores method used to calculate the total score. However, the methodological criterion of validity applies sufficiently for both instruments.

For use in clinical studies, we found the EDSS to be preferred as a primary and secondary outcome measure in recent studies (50 EDSS, 9 MSFC).

**Conclusions:**

Recognizing their strengths and weaknesses, both EDSS and MSFC are suitable to detect the effectiveness of clinical interventions and to monitor disease progression. Almost all publications identify the EDSS as the most widely used tool to measure disease outcomes in clinical trials. Despite some limitations, both instruments are accepted as endpoints and neither are discussed as surrogate parameters in identified publications. A great advantage of the EDSS is its international acceptance (e.g. by EMA) as a primary endpoint in clinical trials and its broad use in trials, enabling cross-study comparisons.

## Background

Multiple sclerosis (MS) is a chronic inflammatory disease of the central nervous system that mainly affects young adults. The disease is characterized by the occurrence of relapsing neurological deficits that affect different functional systems of the central nervous system. The majority of patients initially present with a relapsing remitting disease course (RRMS > 80%), however after 10 – 15 years about 60% of these patients show a transition into a secondary progressive disease course (SPMS) that is characterized by a gradual decline of neurological function. In less than 15% of cases, the disease course is progressive from the onset (primary progressive MS, PPMS) [1].

In recent decades, a number of instruments have been developed that describe the clinical severity and the functional deficits in multiple sclerosis. These instruments are increasingly used as an endpoint in clinical trials to assess the effectiveness of therapeutic interventions.

The most popular and widely used instrument is the Expanded Disability Status Scale (EDSS) of Kurtzke [2]. The EDSS is a clinician-administered assessment scale evaluating the functional systems of the central nervous system. The EDSS is used to describe disease progression in patients with MS and to assess the effectiveness of therapeutic interventions in clinical trials. It consists of ordinal rating system ranging from 0 (normal neurological status) to 10 (death due to MS) in 0.5 increments interval (when reaching EDSS 1). The lower scale values of the EDSS measure impairments based on the neurological examination, while the upper range of the scale (> EDSS 6) measures handicaps of patients with MS. The determination of EDSS 4 – 6 is heavily dependent on aspects of walking ability.

Another important instrument is the Multiple Sclerosis Functional Composite (MSFC) [3], which was developed by the MS Society’s Clinical Assessment Task Force [4] as an additional clinical measure of MS disability progression. The primary goal for creating the MSFC was to improve the standard measure of MS disability for clinical trials and to develop a multidimensional metric of overall MS clinical status [4]. The MSFC is a three-part performance scale for evaluating the degree of impairment in MS patients. It includes the assessment of leg function by moving a short walking distance (“Timed 25-Foot Walk”, T25FT), the assessment of arm function using breadboard test (“9-Hole Peg Test”, 9HPT) and an attention/concentration test to assess cognitive functions (“Paced Auditory Serial Addition test”, PASAT). An integrated MSFC score is calculated using z-scores. There is an ongoing debate about which dimensions to include in the MSFC (e.g. the inclusion of a vision testing) as well as how the reference population affect the standardized scoring (z-scores) of the MSFC [5-13]. In recent years, the MSFC is increasingly used in clinical trials.

A number of other instruments are available to assess MS: the Ambulation Index (AI) [14], the Scripps Neurological Rating Scale (SNRS) [15] and the Illness Severity Scale (ISS) [16], the Guy’s Neurological Disability Scale (GNDS) [17] and the Multiple Sclerosis Impairment Scale (MSIS) [18]. Furthermore, the overall Functional Independence Measure (FIM) [19] and the Cambridge Multiple Sclerosis Basic Score (CAMBS) [20] are clinical assessment instruments that could be used to assess MS [21]. Specific instruments for measuring health-related quality of life in MS patients are the Multiple Sclerosis Quality of Life-54 (MSQOL-54) [22] and the Multiple Sclerosis Quality of Life Inventory (MSQLI) [23]. Only a few of these instruments meet the requirements of methodological standards (e.g. validity, reliability, responsiveness), particularly for use in clinical trials. None of these instruments is recognized to use in clinical trials without any restrictions.

The aim of this study was to identify strengths and weaknesses of EDSS and MSFC. We investigated to what extent the psychometric properties of the two most important instruments – EDSS and MSFC – meet the methodological standards and what value they have in clinical trials. Although different methodological characteristics of the EDSS are the focus of many studies, a summary and synthesis of the study results is missing. In addition, the importance the MSFC is still a matter of debate and a summary of its methodological characteristics is also missing. We addressed this gap by conducting a systematic literature review to specifically answer the following questions:

• Are the instruments suitable to detect the efficacy and/or effectiveness of clinical interventions and to monitor the disease progress?

• Do the methodological properties of EDSS and MSFC meet the required standards of objectivity, reliability and validity as applied to instruments for assessment of disease impact in MS?

• What is the evidence of the practicality and sensitivity to change of these instruments?

• Which methodological shortcomings of EDSS and MSFC must be considered in interpretation of study results?

• Which minimally important difference (minimal clinically important difference, MCID) is reported for EDSS and MSFC?

## Methods

The study was conducted in two phases. First, an exploratory electronic literature search was undertaken to (a) develop and optimize a systematic search strategy and (b) estimate the extent and quality of available studies. Then, a systematic literature search was conducted using the developed search algorithms. The databases MEDLINE (PubMed), ISI Web of Science, EMBASE, PsycINFO and CINAHL & PSYNDEX were systematically searched. Search terms were: *Multiple Sclerosis + Expanded Disability Status Scale OR EDSS* (for EDSS) and *Multiple Sclerosis + Multiple Sclerosis Functional Composite OR MSFC* (for MSFC) (Table 1).

**Table 1:** Results of systematic literature research*

	**EDSS**	**MSFC**	**EDSS or MSFC**	**EDSS and MSFC**
MEDLINE (PubMed) (all limits)	1814	164	1863	115
ISI Web of Science (limit language)	2358	384	2543	208
EMBASE (all limits)	1890	148	1932	106
PsycINFO & PSYNDEX (limits humans, adult)	1282	79	1294	67
CINAHL (limit humans, adult)	504	41	510	35

*According to database limitations, the search was specified by additional search criteria: 1) humans, 2) adult (>18 years old), 3) language (English, German, French, Spanish).

Overall, 3680 results were identified (4272 of the initially generated 8132 hits were excluded as duplicates); the abstracts were reviewed by two independent scientists and structured according to the following criteria:

1) Psychometric studies: validity, reliability, sensitivity of change, head-to-head comparisons, further developments of EDSS or MSFC and reviews.

2) Publications on clinical trials in which the EDSS and/or the MSFC is used as outcome measure or part of it.

3) Other types of studies, if these relate to the central question (for example, review articles).

When the theme did not meet the research question, the abstract was excluded. A total of 684 publications were classified as relevant and ordered as full-text publications in electronic copy or selected in lending (psychometric publications n = 295; papers on clinical studies n = 364; reviews, summaries etc. n = 25).

In a further selection process the full-text psychometric papers (n = 295) were reviewed and subdivided into:

1) Studies that include information about the psychometric properties of the investigated scores (validity, reliability, sensitivity to change, and head-to-head comparisons).

2) Studies that focus on a different instrument than EDSS and/or MSFC (without acceptable psychometric information about the EDSS or MSFC).

3) Studies that are not relevant to the central study questions.

**Figure 1:**
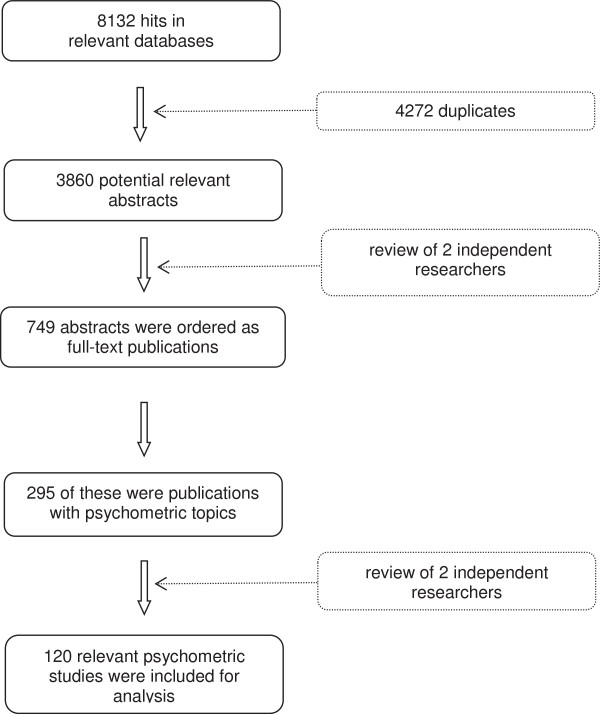
Selection process of methodological publications for EDSS and/or MSFC.

The categories 2 and 3 were excluded. Ultimately, 120 papers were identified as relevant methodological publications. Figure 1 gives an overview of the selection process.

In addition, the publications on clinical trials were reviewed. Furthermore, the public register of clinical trials *ClinicalTrials.gov* by the U.S. National Institutes of Health was systematically searched and an overview of relevant clinical studies generated.

## Results

We identified 120 publications with methodological content for EDSS and/or MSFC. Table 2 gives an overview of scores distribution and period of publication.

**Table 2:** Psychometric publications classified by score and year

**Score**	**Year of publication**			
	**Up to 1999**	**2000-2009**	**Since 2010**	**Total**
EDSS	29	19	6	54
MSFC*	3	19	4	26
EDSS + MSFC* **	2	33	5	40
Head-to-head-comparisons	-	11	1	12
Total				120

*Some studies deal only with parts of the MSFC (T25FW, 9HPT, PASAT).

**This includes although publications that examine other instruments besides EDSS and MSFC.

Numerous relevant studies of EDSS were published before 2000 [24-32]. The majority of the MSFC studies as well as the head-to-head comparisons were published between the years 2000 and 2009.

### Development of EDSS and MSFC as endpoint in clinical trials

In the early clinical studies evaluating therapeutic interventions for MS, the effectiveness of treatment was exclusively measured by reducing the number of relapses, due to the importance of relapses for disease progression [33-38]. However disease progression measured by EDSS was introduced as a primary endpoint in 1996 when the therapeutic effect of Interferon-beta 1a i.m. was evaluated. Currently, progression of disability measured by EDSS is the most important secondary endpoint in MS trials addressing RRMS patients and the most important primary endpoint in all studies dealing with therapeutic interventions in patient with a progressive disease course. Recent studies usually also include the MSFC-score as an endpoint in clinical trials [39-41].

Therefore the current guidelines of the European Medicines Agency (EMA) [42] recommend prevention and delay of disability progression for SPMS and PPMS studies and relapses, disease progression and the increase in disability for studies on RRMS or SPMS (with superimposed relapses) as primary or secondary endpoints.

### Psychometric properties of EDSS and MSFC

#### EDSS

Overall, we identified 54 full-text publications in which the psychometric properties of the EDSS (without comparison to the MSFC) were examined. One of the studies is the original work of Kurtzke [2], 9 were summary papers or reviews, 21 investigated the validity, 13 the reliability, 8 the sensitivity of change and 10 included modifications of the EDSS, especially versions for self-reporting. In addition, there is a simulation study on power (overlaps are possible). (The 40 studies which consider the MSFC as well are not listed here; for more information see MSFC.)

Kurtzke himself did not test the psychometric properties of EDSS. A series of studies on the properties and the functional systems of the EDSS was conducted as awareness of psychometric methods increased [24-29,32,43-45]. Most of the early studies concentrated on reliability. A first study considering reliability, validity and sensitivity to change is the work of Sharrack and colleagues [29]. A detailed study on the validity, reliability (inter-and intrarater reliability), sensitivity to change, and floor and ceiling effects on a sample of patients with MS is published from Hobart and colleagues in 2002 [32].

##### Validity

The validity of the EDSS has been established in numerous studies: Amato and colleagues [31] certify a good validity of the EDSS; Ebers [46] described the EDSS as “well validated”. In early studies, the EDSS was correlated with the Barthel Index (BI), the London Handicap Scale (LHS), the Scripps Neurological Rating Scale (SNRS), the Functional Independence Measure (FIM) [47] and physical function ability of the SF-36; strong to very strong correlation could be shown [29,32,48], weaker results were reported between the EDSS and the ambulation index (AI) [17,49]. In addition, the EDSS correlated with neuropsychological impairment and brain changes measured by MRI – with no or weak correlations [50]. Results of correlations of EDSS with patient-reported outcomes were heterogeneous, from moderate to no correlation.

In studies checking the validity of the MSFC, the EDSS is often used as gold standard reference (see the validity of the MSFC in the next section).

##### Reliability

A criticism of several studies is the limited reliability of EDSS measurements [24,25,27,44,51]. For inter-rater reliability kappa values between 0.32 to 0.76 for the EDSS and between 0.23 to 0.58 for the individual functional systems were reported [24,25,27,44,52]. The intra-rater agreement is slightly higher than the inter-rater, they both show greater variability for lower EDSS scores (1.0-3.5) than for higher score values [27,53].

Different intervals of agreement (exact match, one step (0.5 points), two steps (1.0 points), etc. on the EDSS scale) affect the degree of reliability; studies that attest good reproducibility to the EDSS allow higher deviations. From a deviation of 3 steps on the EDSS scale (1.5 points) almost all studies report good to perfect inter-rater reliability.

##### Sensitivity to change

In the literature review, the EDSS was found somewhat sensitive to changes in disease progression [17,54]. Schwid and colleagues [55] compared limitations of walking ability in MS patients, the EDSS and Ambulation Index and found them to be less sensitive to change than the Dmax (the maximum distance that a person can go) and T8 (time to walk 8 m). Similarly, Vaney and colleagues [56] reported lesser changes in EDSS than in the Rivermead Mobility Index (RMI), the AI and the 10 m walking time test. Hohol and colleagues [53] reported a lower sensitivity to change in EDSS compared with Disease Steps. In turn, Koziol and colleagues [57] observed in a clinical study that the EDSS and the Scripps Neurological Rating Scale could reflect improvements in the cladribine group and deteriorations in the placebo group appropriately.

Moreover, some studies showed that the rates of change varied depending on the initial value. Greater rates of change were observed for minor severity of disease, but from an EDSS score of 6, the EDSS showed very little change in sensitivity [58]. Ravnborg and colleagues [59] reported higher annual rates of change (worsening health) at low EDSS baseline.

##### Feasibility, scale- and distribution properties

The EDSS shows a bimodal frequency distribution in a number of studies [29,30,54,57] with only a few values in the central region and two peaks around the values 3 and 6 [31,53,59].

In numerous studies the unequal interval distances between the EDSS have been criticized, [31,54,60], particularly in the context of clinical trials. This leads to different meanings of change depending on the position on the scale: a difference between the values 1.0 and 2.0 has a different relevance as between 6.0 and 7.0 [61]. Amato and Ponziani [31] as well as Drulovic and colleagues [62] criticized that the use of parametric statistical methods is not possible. The EDSS is also not suitable for use in economic analyses [63]. The non-linearity was demonstrated in studies comparing the EDSS with the health-related quality of life [22,64].

As reported in the literature, the lower scale values (0-4.0) are influenced by impairments detected by the neurological exam of eight functional systems, while the values above 4.0 are mainly based on the walking ability, and values above 6 mainly on patients handicaps [56,62].

##### Interpretation and comparability

A clear recommendation how to interpret changes in EDSS-values cannot be found in the literature. Noseworthy et al. [24] recommend a progression of 1.0 as a meaningful change in clinical trials. Similarly, Healy and colleagues [65] recommended using a continuing progression by one unit on the scale for at least 6 or 12 months. On the other hand, Francis et al. [25] suggested a change of 1.5 points to be more appropriate. For patients with an EDSS of 5.5 or higher at inclusion in the study, progression by 0.5 was deemed sufficient, since changes in this range of the scale are easily perceptible [66].

The guidelines of the European Medicines Agency (EMA) [42] suggest that an average change from baseline is not an adequate efficacy parameter. Instead, they recommend defining treatment success or treatment failure of either reaching a certain EDSS score or a sustained change in sufficient volume. A separate consideration of the lower and upper value range of the EDSS is recommended: 1 point on the EDSS scale with a baseline EDSS score less than or equal to 5.5 and 0.5 points in an EDSS score over 5.5.

A commonly recognized benefit of the EDSS is its good comparability of results from different studies [31,42,63]. The EDSS is fairly robust for measurements over a long period [46,62].

#### MSFC

Overall, we identified 66 full-text publications in which the psychometric properties of the MSFC are reported. Of these, 10 were reviews or summaries, 14 examined the validity, 18 the sensitivity to change and 4 the reliability. Seven publications included modifications to the MSFC. A total of 40 publications also contain information on the EDSS with two publications assessing the superiority of the MSFC over the EDSS [3,4] (overlaps are possible).

##### Validity

MSFC has been shown to correlate with other indicators of disease in MS, including the EDSS, MRI measures (although correlation here is weak) [67,68], patient reported health [69-71] and employment [3,5,72-74]. The predictive validity of MSFC has also been shown for EDSS, brain atrophy, SPMS disease and self-reported health [74].

The EDSS is used most often to test the validity of the MSFC – studies found strong correlations between the two scores, ranging from -0.41 to -0.83 [3,71,75-77]. The PASAT is the weakest correlate [3]. Kalkers and collegues [78] found that while overall MSFC scores distinguished across MS disabilities, the PASAT could not distinguish as well. In fact, Brochet et al. [67] found no correlation between PASAT and EDSS. Rudick and collogues [9] found that MSFC progression seem to be driven by one component of the MSFC, the Timed 25-Foot Walk (T25FWT).

Correlation with MRI measures [67,68] and patient reported outcomes [69-71] were inconsistent, showing moderate to no correlations. For example, MSFC was correlated with the physical component of the SF-36 scale and the sickness impact profile, but not with the mental component of the SF-36 [70]. Change in MSFC score also is shown to have a very weak correlation with change in SF-36 score [79].

##### Reliability

The source publication [4] found MSFC reliability to be high (intra-rater ICC = 0.98, inter-rater ICC = 0.96). Inter-rater and intra-rater reliability were shown to be high for MSFC overall score as well as its component measures. The intraclass correlation (ICCs) were above 0.8 across studies [4,52,78,80]. Schwid and colleges [81] found that MSFC scores varied over measurement periods (over 5 days) but all within 20% change in raw scores.

However, the 9HPT and PASAT have well demonstrated practice effects, meaning that as participants learn about the test, their scores improve [4,52,80,82,83]. This is a general problem of all studies that follow patients over time. The MSFC manual suggests administering the PASAT at least three times prior to baseline measurement to address this practice effect as there is some evidence that practice effects stabilized by the 4th observation [73,75]. However, some evidence point to practice effect persisting beyond fourth testing repeats [83].

Some studies did not detect notable practice effects [71,84,85]. However, due to potential artifacts that may lead to overestimations of improvement in MSFC scores, precautions should be taken during data collection.

##### Sensitivity to change

In the source database [4], MSFC was found to significantly decline from baseline (z = -0.14) to 2 years (z = -0.14) for patients in placebo groups of clinical trials. They also found patients with MSFC deteriorating by at least one standard deviation to have an odds ratio of 2.1 for sustained EDSS deterioration [3,4].

Some studies indicate the MSFC to be more sensitive than EDSS to detecting changes in disease [71,84-86]. However, due to missing dimensions (e.g visual function) there is also evidence that the MSFC is not more sensitive than the EDSS [73,77]. Others found the PASAT does not reflect disease progression in certain MS populations [11 for PPMS].

##### Feasibility

Fischer et al. [4] found that it took 15 minutes for trained personnel to administer the MSFC to patients (using a small sample of 10 patients). The PASAT, however, is notoriously disliked by patients [73]. From our review, some studies found patients not willing to undergo the PASAT [76,87].

##### Interpretability

A major drawback of the MSFC is its interpretability, especially in the scoring mechanism. Although choice of reference population does not affect statistical significance [4], it does change the value of the z-scores, making cross-trial comparison problematic. Some studies examined reference population choice on MSFC scores [10,78,88] and found the question of reference population problematic.

A further objection to z-score interpretability is its abstractness from clinically meaningful values. A solution involves using clinically relevant cut-offs in the raw scores of MSFC dimensions as opposed to z-scores. Bosma et al. [82] found optimal cutoff values to be 20% change for the T25FW and 20% for the 9HPT but could not determine a relevant cutoff for PASAT. Hoogervorst et al. [69] established that 20% change in all three MSFC measures were meaningful to patient’s own perception of health (using the GNDS as a reference measure). Similarly, other studies found the 20% change in T25FW [89,90] and 9HPT [90] reflect disease status in MS patients. Rudick et al. [9] further found, using data from the AFFRIM and SENTINEL trials, that a 15 or 20% change, sustained for 3 months, seems to be an adequate way to quantify the MSFC dimensions. However, others found the 20% not sufficient to detect neurological worsening [81].

### Use of EDSS and MSFC in Clinical Trials

In addition to the review, a systematic search in the public register of clinical trials *ClinicalTrials.gov* by the U.S. National Institutes of Health was conducted to identify relevant clinical trials for MS. Under the topic *multiple sclerosis* (search terms) 904 studies were identified. In a second step, clinical phase III or IV studies on interferon beta-1b, interferon beta-1a, glatiramer acetate, fingolimod, natalizumab, mitoxantrone and cyclophosphamide as well as – due to their current importance – studies on laquinimod, teriflunomide, BG-12 and alemtuzumab were extracted. Finally, 66 clinical phase III or IV studies to a relevant agent of MS therapy could be identified. In 50 of those studies EDSS and/or MSFC were used as primary and/or secondary outcome measurement method (in 15 studies EDSS/MSFC were not reported, in 1 study EDSS was named as a tertiary endpoint). In 9 of the 50 relevant studies the MSFC was used in addition to the EDSS, but there is no study that used only the MSFC (Table 3). It was found that the EDSS has been used preferentially as primary and secondary outcome measurement.

**Table 3:** EDSS and MSFC as primary or secondary outcome in relevant clinical trials*

	**Start of study**		
	**1990 - 2000**	**2000 – today**	**Total**
EDSS	13	37	50
Primary outcome	10	17	27
Secondary outcome	3	20	23
MSFC	-	9	9
Primary outcome	-	1	1
Secondary outcome	-	8	8

*Not taken into account is that the EDSS is in addition to the primary outcome used as secondary outcome. In this overview, only a classification in the “higher” endpoint occurs.

## Discussion

This study examined the extent to which the psychometric properties of the most important instruments to assess disease progression in MS – EDSS and MSFC – meet the methodological standards and what value they have as outcome measures in clinical trials.

In a systematic literature review of 120 relevant full-text publications, we found that while the EDSS is the most widely used instrument in clinical trials assessing the effectiveness of therapeutic interventions, the MSFC is becoming increasingly important.

Single methodological characteristics of the two instruments have been investigated in numerous studies. The EDSS has some documented weaknesses in reliability and sensitivity to change. Although the MSFC was developed rigorously, its weaknesses include interpreting the z-scores (which are used to calculate the summary score from the three components), the learning effects of the PASAT, low acceptance by patients and lack of a visual dimension. However, the MSFC has better sensitivity and reliability compared to EDSS. The methodological quality criterion of validity is sufficiently met by both instruments.

Despite the criticisms, in particular the EDSS could be classified as an important endpoint. In neither publication, the EDSS (or MSFC) are discussed as a surrogate parameter (in contrast to MRI measurements). Cohen et al. [73] emphasized in their review of clinical outcome parameters, the general acceptance of the EDSS as an endpoint by different authorities. In contrast, the MSFC is usually used as a secondary endpoint.

The importance of the instruments is also reflected in our evaluation of clinical trials: in 50 of 66 identified relevant phase-III and phase-IV studies, disease progression as measured by EDSS was used as primary (27 studies) or secondary outcome (23 studies). The MSFC was used in eight studies as a secondary endpoint and only one study as a primary endpoint.

In summary, both instruments are acceptable outcome criteria to assess the effectiveness of clinical interventions and to monitor disease progression. When using the EDSS, its limited inter-and intra-rater reliability should be considered. All possibilities to increase reliability should be used, including training of investigators, assessment by the same doctor/neurologist during the study, specified times of detection, standardized protocols for neurological examination and a precise definition of all requirements. When using the MSFC, the learning effects of the PASAT and 9HPT should be considered and controlled (e.g. using control groups).

Concerning the natural history of MS, Weinshenker and colleagues [1] observed an average change of 0.5 points on the EDSS scale in a year. A clear recommendation on interpreting changes in EDSS and MSFC values does not yet exist. EDSS changes by 1.0 points from a baseline EDSS less than or equal to 5.5 and 0.5 points over a baseline 5.5 are commonly recognized as a clinically increase in disability. However, it is now understood that it is more accurate to define disability change as a sustained change for 12 weeks or, even more reliably, for 24 weeks.

For the MSFC, changes by 20% in the individual components are considered to be clinically relevant. However, it is difficult for a clinician to understand what a 20% change in MSFC subscores is while a significant EDSS change is more intuitively understood.

Overall, the literature reveals that the EDSS is the most widely used and best-known instrument to assess disease progression in MS. The advantages and disadvantages of EDSS and MSFC have been investigated frequently. The great advantage of the EDSS is its international acceptance (including the EMA) as a primary endpoint in clinical trials. Because it is so commonly used, studies that use the EDSS can easily compare results to other findings. Following the current literature, we can conclude that the importance of the EDSS will be unabated in future. Major changes of the EDSS are not recommended, as not to jeopardize its advantage.

In addition, the use of other measurements as clinical trial endpoints (e.g. the MSFC) is recommended to provide information on dimensions no covered in the EDSS, such as upper limb function or cognitive skills.

## Conclusions

The assessment instruments we examined, EDSS and MSFC, both are frequently and internationally used in clinical studies – despite their well know methodological limitations. They are suitable to detect patient-relevant endpoints in MS with reasonable validity in spite of some methodological concerns.

## Consent

Written informed consent was obtained from the patients for the publication of the reviewed studies and accompanying images.

## Competing interests

The authors declare that they have no competing interests.

## Authors’ contributions

SMM conducted the literature review and drafted the manuscript. YSF conducted the literature review and drafted the manuscript. MM drafted the manuscript and gave clinical input. FWD contributed to the clinical studies section and drafted the manuscript. TK conceptualized the study and critically reviewed the manuscript. All authors read and approved the final manuscript.

## Pre-publication history

The pre-publication history for this paper can be accessed here:

http://www.biomedcentral.com/1471-2377/14/58/prepub
